# HBVRegDB: Annotation, comparison, detection and visualization of regulatory elements in hepatitis B virus sequences

**DOI:** 10.1186/1743-422X-4-136

**Published:** 2007-12-17

**Authors:** Nattanan Panjaworayan, Stephan K Roessner, Andrew E Firth, Chris M Brown

**Affiliations:** 1 Department of Biochemistry, University of Otago, Dunedin, New Zealand

## Abstract

**Background:**

The many *Hepadnaviridae *sequences available have widely varied functional annotation. The genomes are very compact (~3.2 kb) but contain multiple layers of functional regulatory elements in addition to coding regions. Key regions are subject to purifying selection, as mutations in these regions will produce non-functional viruses.

**Results:**

These genomic sequences have been organized into a structured database to facilitate research at the molecular level. HBVRegDB is a comparative genomic analysis tool with an integrated underlying sequence database. The database contains genomic sequence data from representative viruses. In addition to INSDC and RefSeq annotation, HBVRegDB also contains expert and systematically calculated annotations (e.g. promoters) and comparative genome analysis results (e.g. blastn, tblastx). It also contains analyses based on curated HBV alignments. Information about conserved regions – including primary conservation (e.g. CDS-Plotcon) and RNA secondary structure predictions (e.g. Alidot) – is integrated into the database. A large amount of data is graphically presented using the GBrowse (Generic Genome Browser) adapted for analysis of viral genomes. Flexible query access is provided based on any annotated genomic feature. Novel regulatory motifs can be found by analysing the annotated sequences.

**Conclusion:**

HBVRegDB serves as a knowledge database and as a comparative genomic analysis tool for molecular biologists investigating HBV. It is publicly available and complementary to other viral and HBV focused datasets and tools . The availability of multiple and highly annotated sequences of viral genomes in one database combined with comparative analysis tools facilitates detection of novel genomic elements.

## Background

Hepatitis B virus (HBV) chronically infects about 350 million people worldwide and is a major contributor to liver pathology including hepatitis and carcinoma. A large number of strains, isolates and mutants of the *Hepadnaviridae *family have been sequenced. For example, a search of Entrez for HBV complete genomes currently (9/2007) retrieves 1114 records, and the Hepatitis Virus Database (HVD) contains over 1000 full-length sequences. The small, just 3.2 kb, genome has been extensively studied – with a PubMed search for 'HBV genome' resulting in over 2500 publications. This research has shown that the genome is highly packed with information in sequence and structure. This directs processes such as transcription, reverse transcription, replication, nuclear import and export and coding [1-7]. Regulatory elements control this at the DNA, RNA and protein levels, with particular bases known to participate in DNA and RNA elements and also encode more than one protein in alternative frames. During infection the mutation rate is high – estimated to be around 10-5 to 10-4 per base per year [8]. This results in a quasi-species infecting a single individual and may result in some DNA sequences from an individual not being representative of the 'fittest' species. Mutants may become prevalent in the population – for example, precore mutations, escape mutations, or antiviral resistance mutations.

Recently several international public databases containing significant hepadnaviral content have become available: the general Viral Reference Sequence genome project [9,10], Hepatitis Virus Database [11], SEQHEPB [12], and the HepSeq database [13]. Each has its own focus and utility. The viral RefSeq genome project is broad but includes 10 *Hepadnaviridae *members. It is searchable through Entrez Genomes and linked to other resources including the protein database, NCBI gMap and gene [10]. The HepSeq database is an epidemiological database focussing on epidemiological, clinical nucleotide sequence and mutational aspects of HBV infection [13]. The Hepatitis Virus Database includes HBV and provides information on genome location and phylogenetic relationships automatically processed from DDBJ [11]. SEQHEPB allows subscribers to analyze genotypes of HBV genomes, including key mutations associated with antiviral resistance [12]. However, there is no tool available to combine expert annotation with similarity search methods for molecular biological research into HBV [14-17].

We describe here a genome-based public domain database for the *Hepadnaviridae*. The database contains data on individual sequences and groups of sequences and facilitates comparative genomic analysis. The complexity of the HBV genome has challenged development of this resource but it will provide a model for other viruses.

## Methods

### Sequences for analysis

For more detail refer to the documentation in the database. Genome sequences of selected representative viruses of the *Hepadnaviridae *family were retrieved from NCBI. All retrieved Genbank files were split into fasta-formatted and gff-formatted files. As the virus genomes are circular, some of the parsed Genbank files were manually curated in order to be represented correctly.

### Processing of data

Multiple sequence alignments were produced with ClustalW [18]. All files were then placed in the MySQL database HBVRegDB.

To identify conserved viral genomic regions, three blast queries (blastn, tblastx and blastx) were performed on RefSeq Virus release 24 with the parameters shown in Table 1. The results were reformatted to create a gff file and the names of the matched sequences were integrated to present them in a meaningful graphical representation. The database will be updated with RefSeq releases.

**Table 1:** The blast parameters used to perform the BLAST queries.

Parameter	Meaning	blastn	tblastx
-e	Expectation value E	100 (10.0)	10(10)
-q	Penalty for nucleotide mismatch	-1 (-3)	-
-r	Reward for nucleotide match	1 (1)	-
-E	Cost to extend a gap	-1 (-2)	-2(1)
-G	Cost to open a gap	-2 (-5)	-8(11)
-W	K-tuple size	7 (11)	2(3)

RefSeq Virus was chosen as the target database as the redundancy in Genbank makes the top matches less informative. The penalty for a mismatch, the cost to open and to extend a gap has been specifically set to detect distant similarity matches.

BLOSUM62 was used for tblastx.

## Results and discussion

### Annotations on single viral genomes

**Figure 1:**
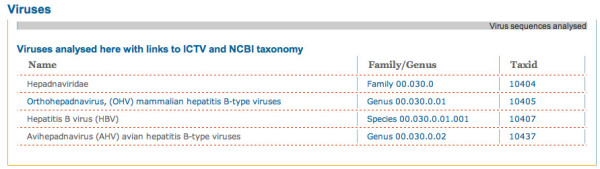
Screenshot of a table indicating genomic sequences analyzed in HBVRegDB.

The NCBI viral RefSeqs for *Hepadnaviridae *provide the best information about coding regions and protein sequences. However, in general they do not provide information on regulatory signals that are crucial for viral gene expression. Four NCBI taxonomic groupings (Figure 1) were incorporated into HBVRegDB.

Eight HBV genotypes (A-H) have been described that vary in up to 15% of bases. They have very similar genomic organization, but differing global prevalence. Infection has been suggested to result in different clinical outcomes, and this is presumably related to sequence variation [19-25]. Other HBV-like viruses also infect hominids (Hominid HBV, HHBV, ~20% nt divergence from HBV) and rodents (*Orthohepadnavirus*, OHV, ~45% nt divergence from HBV). Closely related viruses infect birds (*Avihepadnavirus*, AHV) with overall similar organization but significant sequence divergence (~60%). These have been used as models to investigate human HBV [26].

**Figure 2:**
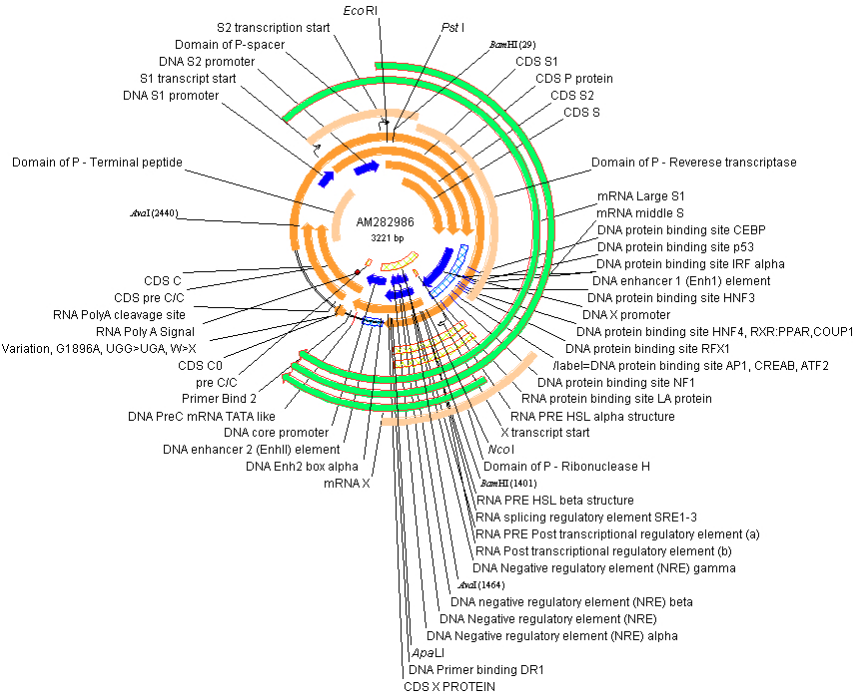
The highly annotated reference sequence (AM282986) in Genbank format visualized by VectorNTI.

As part of our experimental research, a complete HBV genome *adw*, genotype A, derived from a Taiwanese HBV-infected patient was sequenced (a gift from M-H Lin, National Taiwan University). This HBV clone was known to produce viable HBV particles when transfected into cells [27]. This sequence was highly annotated and submitted to INSDC via EMBL (EMBL ACC: AM282986; Figure 2). The annotation was done by extracting biological information from the literature or other sequence records, based on the functional conservation. This sequence has the most complex annotation per nucleotide (3–13 annotations per base) of any sequence in the public sequence database. It represents the limits of this type of annotation and of parsers implemented to interpret it. It was intended to annotate most experimentally proven features on the sequence. This will lead to annotation of features that may be of lesser importance (e.g. the S protein myristoylation site [28]) or alternative splicing [29,30].

Sequences were numbered to begin at the EcoRI site position (if present). This was chosen because, for most sequences, the numbering is common until base ~1910, where the numbering diverges. An alternative logical numbering scheme is also used from position 1 in the pregenomic RNA [31]. Protein sequences are separately described using the standard numbering [32].

#### Annotated DNA elements

Two Direct Repeat DNA primer-binding sites – DR1 and DR2 – are involved in replication and may be preferential sites for viral integration into the host genome [4]. Promoters – preC/C promoter and TATA box [33], S1 promoter, S2 promoter, X promoter. Transcriptional regulatory elements – NREα, NREβ and NREγ. (reviewed in [4]. Enhancers – Enh I and Enh II, which modulate mRNA synthesis. The functions of Enh1 and EnhII were demonstrated for the HBV *ayw *subtype [4]. Protein binding sites. DNA binding sites within the central core domain of Enh I. Binding sites of C/EBP, p53, IRFα, HNF3, HNF4, RFX1, AP1, NF1, CREAB, ATF2, RXR:PPAR and COUP1 (reviewed in [34]. An element within Enh II, box α, which is essential for function of the enhancer *in vivo*. The non-canonical polyadenylation (TATAAA) signal used by all transcripts [33,35] followed by the poly (A) cleavage site (nucleotide 1930). An indicative variation which represents a nucleotide transition from 'A' to 'G' at nucleotide position 1896 changing a preC tryptophan to a termination codon [36]. There are many functional and non-functional variants of HBV and it is not the focus of this database to show them; this is done by existing databases – e.g. HepSEQ and SEQHEPB [12,13].

#### Annotated RNA elements

Five mRNAs – preC, pgRNA, S1, S2, and X, all ending at the common poly (A) cleavage site including alternative splice variants of these transcripts [27,37]. RNA regulatory elements – post-transcriptional regulatory element (PRE; reported to be an important RNA export element [38-40]), splicing regulatory element (SRE) 1–3 [41], conserved stem-loop structures within the HBV PRE, PRE HSL α, PRE HSL β [38], and the critical RNA epsilon element structure required for replication and packaging [42].

#### Annotated protein coding sequences

Eight CDS were annotated on the sequence – preC, C, P, X, large S, middle S, small S and C0. C0 is a small CDS not annotated on most HBV genomes. It is involved in regulation of translation of the P and C CDSs and is conserved in all HBV genotypes [31]. Protein domains: P – Terminal protein, Spacer, Reverse transcriptase, RNase H. [32].

This highly annotated nucleotide sequence can be downloaded from HBVRegDB in formats designed for use in software that will read Genbank format. A number of the most sophisticated parsers were tested by directly retrieving the entry from an INSDC database (NCBI Genome Browser, Artemis, Apollo (free), VectorNTI (free for academics)). These had differing levels of ability to represent complex annotation, with features (e.g. the P CDS) crossing the origin of a circular genome and complex descriptors (e.g. mRNA, alternative splices) parsed more or less well. In HBVRegDB we provide two slightly modified annotations of this HBV genome. One for more accurate circular parsing into VectorNTI, and another for linear browsers (e.g. GBrowse, Argo). A graphical representation of this annotated sequence in VectorNTI is shown in Figure 2. Although it can represent circular genomes, this format becomes difficult to interpret with many annotations.

HBVRegDB provides a tool to map these annotations onto another HBV sequence by performing a pairwise alignment.

#### HBV, rodent and avian hepadnaviral RefSeq genomes

Key additional regulatory elements were added to HBV genotype C (RefSeq NC_003977), Woodchuck RefSeq (WHV; NC_004107) and Duck HBV RefSeq (NC_001344). These modified sequences are indicated by 'm' e.g. NC_003977m. The additional features for WHV include woodchuck post-transcriptional regulatory element (WPRE), which is reported to enhance gene expression delivered by retroviral vectors for gene therapy [43], and WREα, WREβ and WREγ, whose sequences are conserved within the mammalian hepadnaviruses and are essential for WRE function [44].

### Annotations on multiple sequence alignments

#### HBV_HBVRegDB_32

**Figure 3:**
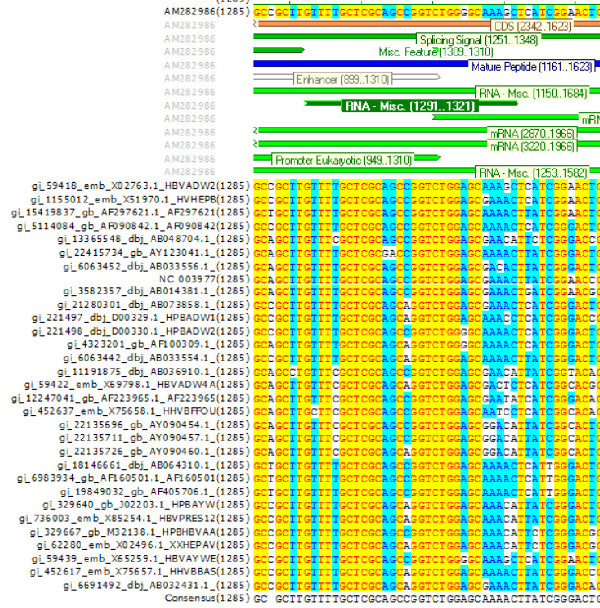
**Multiple sequence alignment of HBV_HBVRegDB_32**. The figure shows the region of the conserved RNA secondary structure known as HBV SLα (nucleotide 1292–1321, [38]). The annotation of genotype A (AM282986) is shown.

This is an annotation of the 23 NCBI genotyping sequences, with other members of genotypes B-F added from [32]. The most highly annotated sequences: NC_003977m RefSeq (genotype C) and AM282986 (genotype A) are included in the alignment. This alignment is available in VectorNTI format (apr) with annotation (e.g. Figure 3) and also in formats that cannot be automatically annotated (msf and aln).

#### HHBV_HBVRegDB_21

Hominid HBV. This is an alignment of human, gibbon, gorilla, chimp and orang-utan HBV genomes from [17], NC_0003977m group.

#### OHV_HBVRegDB_12

*Orthohepadnavirirus *(OHV). This is an alignment of primate and rodent HBV genomes, NC_003977m group.

#### AHV_HBVRegDB_5

*Avihepdnavirirus*. This is alignment of avian HBV genomes, NC_001344m group

### Web-based graphical representation using GBrowse

A set of 65 representative HBV sequences from these groups of alignments is available using Gbrowse. For HBVRegDB the GBrowse software package was chosen because of its flexible configuration and efficient handling of large amounts of data, although a limitation here is the lack of ability to represent circular genomes. Annotations of conserved elements consist of large amounts of data, e.g. more than 30,000 records for one viral genome. GBrowse uses a Bio::DB::GFF schema in a MySQL database and a fetch request is answered by the database query engine in a satisfactory time of ~20 seconds.

### Underlying MySQL database

**Figure 4:**
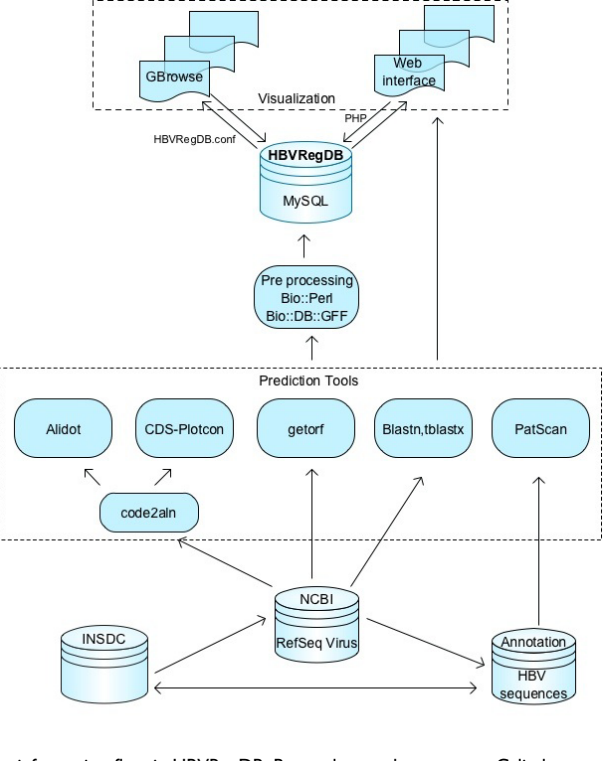
Schematic overview of the information flow in HBVRegDB. Boxes denote data sources. Cylinders represent database components.

A version of MySQL was installed and configured for GBrowse. A Bio::DB::GFF schema was created and integrated into HBVRegDB to store the virus genome sequence data, annotations, statistical track data, and textual information. The five core tables of the HBVRegDB MySQL database contain additional taxonomic information, virus group relationships, web page search related data, and a comprehensive link reference list. An overview of the entire application information flow is shown schematically in Figure 4.

### Statistical and similarity search annotations on single sequences

#### Potential protein coding regions

**Figure 5:**
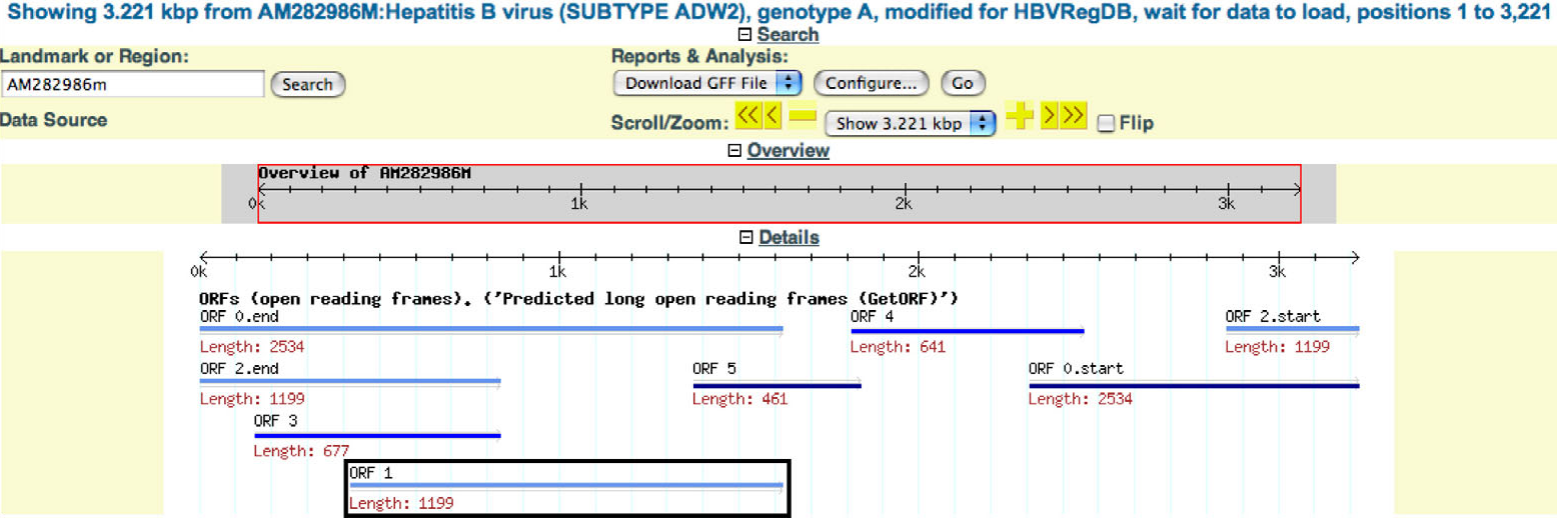
**The top part of the screenshot showing annotations of the AM282986m Hepatitis B virus genome**. Calculated ORFs are represented as bars. This analysis indicates ORFs that could potentially be initiated at different ATG codons. For example, the predicted nested ORF 1 (marked by box).

Where annotated, CDSs are shown. For consistency, and for HBV genomes for which not all CDS sequences are annotated, potential ORFs >100 aa were calculated with getorf (EMBOSS). Coding regions, which extended over the virtual end of the viral genome sequence were automatically assigned and represented as two parts (e.g. Figure 5). This process also shows ORFs that could potentially be initiated at different ATG codons. For example (Figure 5) the predicted S ORFs, ORF 2 and 3, for which there is experimental evidence, or the predicted nested ORF 1, which could arise by internal initiation within P.

#### Similarity searches against other viral genomes

**Figure 6:**
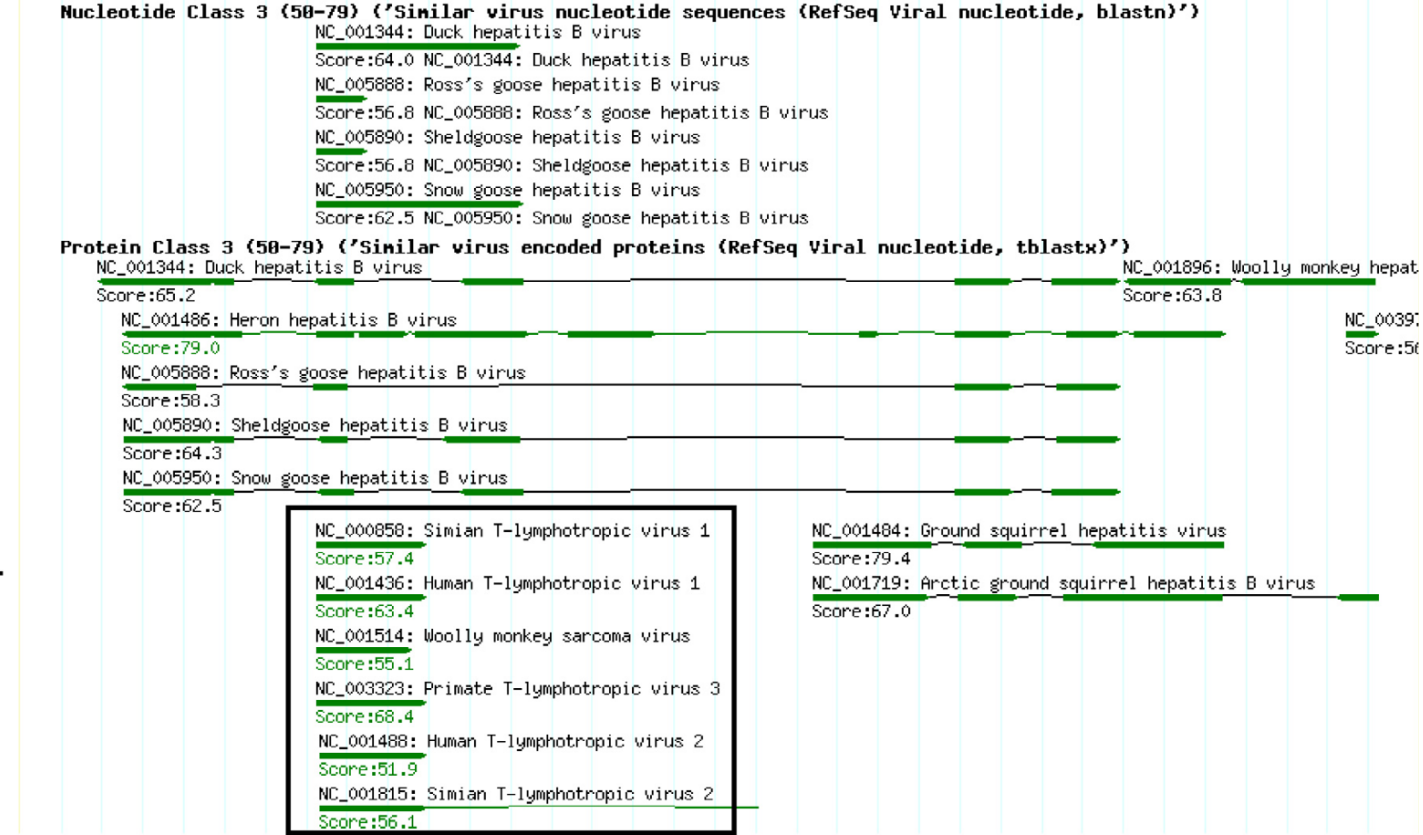
**HBVRegDB-formatted results of the blastn and tblastx query (AM282986) against all viral sequences from RefSeq**. Blast results from HBVRegDB are grouped in different classes based on match scores. This figure displays the results from Class 3 (scores between 50–79). Notably, parameters used in HBVRegDB are adjusted to allow matching of short sequences (-W 2 -G 8 -E 2). For example, the tblastx of HBVRegDB returns the hit of the short motif (YMDD) of the HBV P protein to the YMDD of the P protein from Human T-lymphotropic virus, Simian T-lymphotropic virus 1 and Woolly monkey sarcoma virus (boxed).

Blastn and tblastx were used to detect distant sequence similarities using selected parameters, shown in Table 1. The blastn parameters chosen will detect short exact matches. Tblastx was used to search a six-frame translated sequence against the protein database with hits of greater than two similar amino-acids analyzed. This could identify novel coding regions in query sequences, along with the CDS-plotcon analyses or alternative approaches [14]. Matches are shown in Figure 6. Blastn mainly finds other *Hepadnaviridae*, whereas tblastx (with these parameters) is able to detect more dissimilar matches, e.g. matches between reverse transcriptases from HBV and retroviruses (boxed).

#### Specific regulatory elements

As an example, PatScan [45] as implemented in Transterm [46], was used to identify polyadenylation sites by searching the corresponding pattern AUWAAA. The output files were parsed and gff-formatted files were created and uploaded into the database.

#### User-added custom tracks

The user can add tracks in gff format. The search procedure above can be followed using online tools to annotate any motif that can be described by a regular expression, RNA descriptor or matrix. A description of this procedure is provided on the website.

### Conserved primary and secondary structural elements

**Figure 7:**
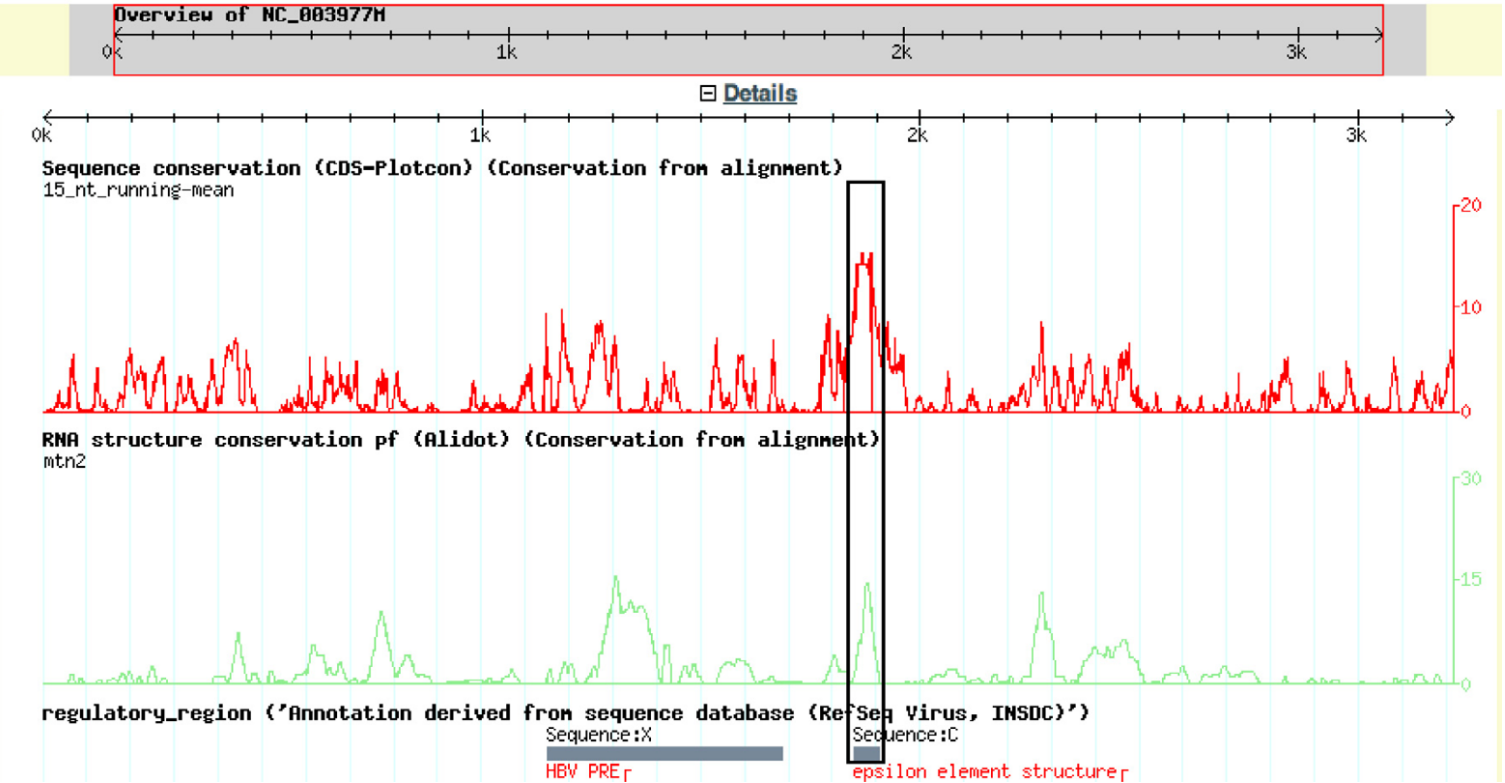
**Conservation analyses (CDS-Plotcon and Alidot) of the virus group with the reference sequence NC_003977**. For example, the known epsilon element is predicted to have conserved primary sequence (CDS-plotcon) and conserved secondary structure (Alidot), as experimentally demonstrated.

A way to detect functional elements in genomes is to look for conserved columns in multiple sequence alignments. However, many columns of CDSs show conservation due to constrains on the encoded protein. CDS-Plotcon is specifically designed to look for conserved functional elements within CDSs, independent of the protein coding constraints. To find conserved RNA structures, the program Alidot [47] was used. For each multiple sequence alignment, the Alidot and CDS-Plotcon results were gff-formatted and uploaded into the database. An example is shown in Figure 7. CDS-plotcon predicts unusually high conservation (higher than that required by the coding capacity in, for example, the boxed region). Similarly the predicted RNA secondary structure (Alidot) has higher than expected conservation in this region. This predicted region is the epsilon element, which is highly conserved in structure and function and is required for viral replication.

### Web interface

All virus genomes in the database can be queried by browsing a table in which information about grouping, genotype, virus name and links to NCBI and to the graphical visualization of the sequence including annotations are provided. There is a comprehensive list of links to related web sites, which is intended to complement research using HBVRegDB. Tutorials in the form of web pages guide users through common analyses, such as:

- Comparison of your sequence to a well-annotated HBV genome.

- Testing for conservation of a sequence across genomes.

- Testing for conservation of an RNA secondary structure across genomes.

- Repeating similarity searches against HBVRegDB Sequences, RefSeq viral genomes and proteins.

## Conclusion and future studies

Focused public domain viral databases have been developed, particularly for HIV, HCV and influenza, but for most viruses this is not available. Part of the approach described here can be generalized to any viral genomes. A preliminary analysis of all ~4000 viral segments in RefSeq has been done, building on the HBVRegDB database, and a comparative viral database (CompVirusDB) is being developed

## Authors' contributions

NP carried out the annotations on single viral genomes, multiple sequence analysis, design of basis for HBVRegDB (e.g. content and structure) and drafted the manuscript. SKR developed web interface and a comparative genomic analysis tool with an integrated underlying HBV viral database. AEF developed a CDS-plotcon programme for detecting functional elements within coding regions. CMB substantially contributed to conception and design of the HBVRegDB, analysis of similarity searches against other viral genomes and preparation of the manuscript. All authors read and approved the final manuscript.
